# Pain Perception of the First Eye versus the Second Eye during Phacoemulsification under Local Anesthesia for Patients Going through Cataract Surgery: A Systematic Review and Meta-Analysis

**DOI:** 10.1155/2019/4106893

**Published:** 2019-06-23

**Authors:** Chuying Shi, Jinqiu Yuan, Benny Zee

**Affiliations:** Division of Biostatistics, Jockey Club School of Public Health and Primary Care, Faculty of Medicine, The Chinese University of Hong Kong, New Territories, Hong Kong

## Abstract

**Background:**

Phacoemulsification under local anesthesia is regarded as the major surgery for cataract treatment. Recent research has compared the pain perception between the first eye and the second eye during phacoemulsification. However, these studies have also yielded controversial findings. Consequently, we performed a systematic review and a meta-analysis to investigate the difference in the pain perception between the first and second eyes during phacoemulsification.

**Method:**

We searched the PubMed, EMBASE, and Cochrane CENTRAL databases for the studies published up to October 5, 2018. Prospective observational studies were included. The meta-analysis was conducted by means of random-effects model and fixed-effects model according to the heterogeneity. Evaluation of the methodological quality of studies was based on Newcastle-Ottawa Scale (NOS).

**Results:**

Overall, eight studies were included in the meta-analysis. The analysis of pooled data showed that the pain scores of the first eye shortly after surgery under local anesthesia were significantly lower as compared to the second eye (WMD: 0.69; 95% CI: 0.40, 0.98; *P* < 0.00001). The average pain scores of the first eye shortly after surgery under the topical anesthesia were also lower than those of the second eye (WMD: 1.08; 95% CI: 0.79, 1.36; *P* < 0.00001). Conversely, anxiety scores in the first eye surgery were significantly higher than those in the second eye surgery (SMD: −0.40; 95% CI: −0.64, −0.16; *P*=0.001). However, the difference of the pain scores accessed on the first postoperative day between the first and second eye surgeries (WMD: −0.05; 95% CI −0.40, 0.31; *P*=0.79) as well as cooperation grades of patients between the first and second eye surgeries (WMD: 0.35; 95% CI −0.07, 0.76; *P*=0.10) was not statistically significant.

**Conclusion:**

Patients experienced more pain in the surgery of the second eye than that of the first eye, which probably related to lower anxiety before the second surgery. It suggests that we should consider preoperative intervention to reduce the perceived pain during second eye cataract surgery.

## 1. Introduction

Phacoemulsification with implantation of a foldable intraocular lens is a common methodology applicable in the cataract surgery as a relatively safe procedure for patients. The method can be delivered in a comfortable surgical atmosphere and induce a relatively low stress level to patients under local anesthesia.

However, phacoemulsification with local anesthesia, including topical anesthesia, intracameral anesthesia, and sub-Tenon's anesthesia, among others, is not a completely pain-free procedure. Pain level is possible and differs in various stages of phacoemulsification. Additionally, most of the pain experienced during an uneventful surgery was reported when the anterior chamber was extended by irrigation [1, 2]. In terms of clinical considerations, some ophthalmic surgeons realized that patients generally have complaints about the unpleasantness and discomfort for the second eye surgery. Previous studies have been performed because of this consideration. In particular, recent studies have shown that the second eye during phacoemulsification was more painful [3, 4]. However, others indicated no difference [5, 6]. As a result, the question of whether the second eye during phacoemulsification is more painful remains a controversial issue even today.

As far as we know, there is no meta-analysis dealing with this topic. Therefore, we carried out a systematic review and a meta-analysis to examine whether the second eye was more painful during the uneventful phacoemulsification under local anesthesia, and to determine risk factors that influence pain perception. In a case where the result has a statistical difference, surgeons can pay more attention to the patients' perception before or during the second cataract surgery to lower the risk of the complication. Such an intervention can also help to improve clinical performances and reduce health costs.

## 2. Materials and Methods

### 2.1. Search Strategy

We searched the database of PubMed, EMBASE, and Cochrane to obtain eligible studies published up to October 5, 2018. Taking PubMed research as an example, following groups of keywords or medical terms were used: “Phacoemulsification” OR “Phacoemulsifications” OR “Cataract Surgery” OR “Phaco Surgery”) AND (“Cataract” OR “Cataracts” OR “Lens Opacity” OR “Lens Opacities” OR “Opacities, Lens” OR “Opacity, Lens” OR “Cataract, Membranous” OR “Cataracts, Membranous” OR “Membranous Cataract” OR “Membranous Cataracts” OR “Pseudoaphakia” OR “Pseudoaphakias”) AND (“Eye Pain” OR “Eye Pains” OR “Pain, eye” OR “Pains, Eye”. In addition, we carried out a manual search of the references of associated studies to include more studies. We constrained the languages to English. This study has been registered at PROSPERO with registration number CRD42018111948.

### 2.2. Study Selection

The following selection criteria were used to identify the published studies for inclusion in this meta-analysis: First, we used prospective observational studies or cohort studies; second, trials counted on the cataract patients who went through phacoemulsification under local anesthesia; third, the trials compared pain scores by visual analogue scale (VAS) between the first and the second eyes. Last, the time of accessing the pain was the day of the surgery or the first postoperative day. In the meantime, the exclusion criteria were as follows: We excluded the trials that have not assessed pain scores or that the data were not available.

### 2.3. Data Extraction

The information provided herein was obtained from the selected studies, in which the following aspects were taken into consideration: every study's first author, publication year, study location, mean age of patients, measurement of outcome for pain and anxiety, scores of pain and anxiety, total numbers of surgery eyes, numbers of first and second eyes, and blind method, among others. In cases of disagreements on the items, a consensus was developed through the discussion among the research groups.

### 2.4. Quality Assessment

Two independent investigators carried out the reviews of study titles as well as abstracts, focused on retrieving studies that meet the inclusion criteria for the full-text evaluation. Subsequently, we conducted analyses of the trials for comprehensive analysis as well as data extraction with an agreement value (*κ*) of 98%; the differences were alleviated by use of a third investigator. Evaluation of the methodological quality of eligible comparative studies was undertaken based on Newcastle-Ottawa Scale (NOS) [7].

### 2.5. Statistical Analysis

We used Review Manager 5.3 (Cochrane Collaboration, 2014) to carry out statistical analysis. For continuous measurements, we used the weighted mean difference (WMD) and standard mean difference (SMD) with 95% confidence interval (CI) as the effect estimate. Moreover, *I*^2^ was regarded as the evaluation of heterogeneity among studies [8], followed by division into three levels: low < 25%, medium 25–75%, and high ≥ 75% [9]. When *I*^2^ amounted to more than 50%, we utilized the random-effects model. Otherwise, we applied the use of the fixed-effects model. We estimated the mean deviation (MD) and standard deviation (SD) from the median, range, and the size of a sample which had been estimated by the ascertained calculation methodology [10]. We further carried out an evaluation of publication bias of studies with the help of Funnel plots and Egger test by STATA 15.1. Additionally, we assessed the impact of each individual study through the exclusion of the studies one at a time.

## 3. Results

### 3.1. Selection of Studies

**Figure 1:**
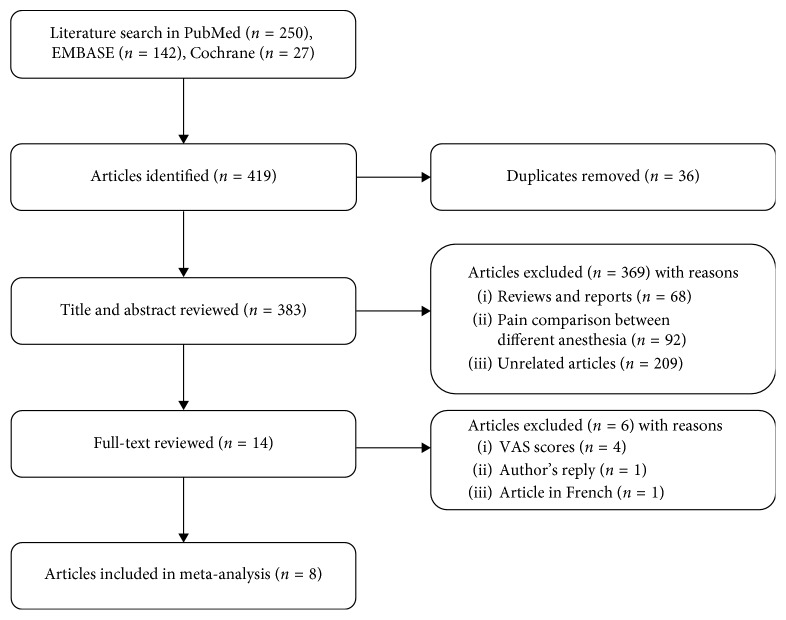
Study selection process.

The progress of the systematic studies search has been displayed in Figure 1. With an initial search, we included 250 records from the PubMed, 142 records from the EMBASE, and 27 records from the Cochrane. An aggregate of 383 articles was acquired after the removal of duplicates. Subsequent to the reviews of titles, abstracts, and full texts, as well as eight studies satisfying our inclusion criteria, were included in the final meta-analysis.

### 3.2. Study Characteristics

The selected eight studies were published between 2008 and 2018 [3–6, 11–14]. A summary of the included characteristics of the studies is shown in Table 1. Two studies were conducted in Australia, two studies in Turkey, two studies in China, one study in USA, and one study in UK. The sample size was in the range of between 38 and 268. Six studies carried out the assessment of the pain shortly after the surgery, one study assessed the pain on the first postoperative day, and one study assessed the pain on both of the days. Five of eight studies merely used the topical anesthesia. Among the remaining studies, one made use of topical anesthesia in combination with intracameral anesthesia, one operated hydrodissection with anesthesia, and one used sub-Tenon's anesthesia. Phacoemulsification was carried out by the same surgeons individually in seven out of eight studies. Summary of samples of included studies is exhibited in Table 1. Since the included studies were not the classic cohorts, the score of quality based on NOS is exhibited in Table 2. All of the studies had a quality score ≥5, which showed a high quality.

### 3.3. Meta-Analysis

**Figure 2:**
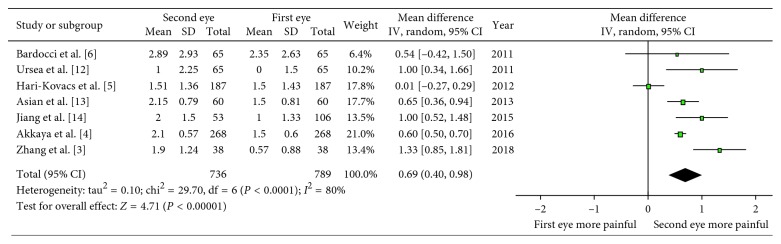
Forest plot comparing the pain scores shortly after the surgery.

The meta-analysis of seven studies of comparing the pain scores assessed shortly following the phacoemulsification under the local anesthesia showed that there were statistical differences in pain values between the first and the second eyes (WMD: 0.69; 95% CI: 0.40, 0.98; *P* < 0.00001; Figure 2) with high heterogeneity (*I*^2^ = 80%). The pain value was lower in patients who underwent the first eye surgery. As demonstrated excluding one study at a time, the heterogeneity is still kept at high level and overall effect showed no obvious change. Therefore, we concluded that no single study markedly affected the overall pooled prevalence estimate. Sensitivity analysis showed the consistent result (WMD: 0.65; 95% CI: 0.33, 0.96; *P* < 0.0001) by dropping the study with quality score less than 7, which suggested the robust outcome.

**Figure 3:**
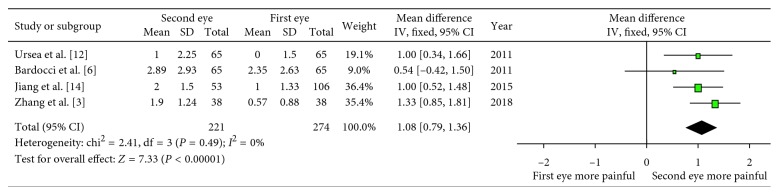
Forest plot comparing the pain scores under topical anesthesia.

We also carried out an analysis of the pain scores only under the topical anesthesia as one of the subgroups. Four of seven studies were included, which also showed a difference (WMD: 1.08; 95% CI: 0.79, 1.36; *P* < 0.00001; Figure 3) with low heterogeneity (*I* = 0%) compared with the high heterogeneity of the comparison under the local anesthesia, which may explain the source of heterogeneity. Because the rest of three studies used different anesthesia, they could not be included into one subgroup. Sensitivity analysis also showed the consistent result (WMD: 1.12; 95% CI: 0.76, 1.48; *P* < 0.00001) by dropping the study with quality score less than 7.

**Figure 4:**
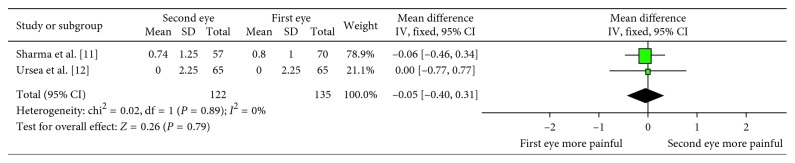
Forest plot comparing the pain scores on the first postoperative day.

**Figure 5:**
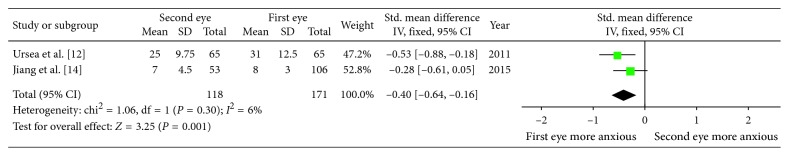
Forest plot comparing the anxiety scores.

**Figure 6:**
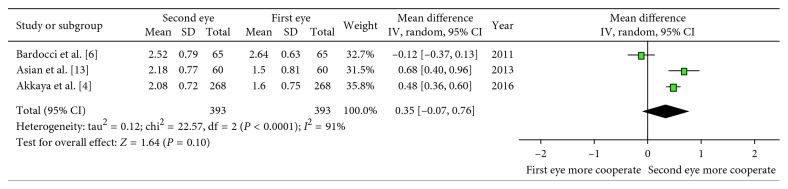
Forest plot comparing the cooperation of patients.

In two studies, pooled data of pain scores on the first postoperative day did not show a significant difference (WMD: −0.05; 95% CI: −0.40, 0.31; *P*=0.79; Figure 4) with low heterogeneity (*I*^2^ = 0%). Three studies compared the anxiety values between the first and the second eye surgeries (Table 1). Due to the longer State-Trait Anxiety Inventory (STAI) survey (administration time 2 to 4 minutes), there was more sensitivity as compared with the Amsterdam Preoperative Anxiety and Information Scale (APAIS) (30 to 60 seconds) in detecting decreased preoperative anxiety [12]. We excluded one study because it assessed the anxiety on the day after surgery other than before the surgery. Finally, two studies by STAI were included. There was a statistical significant difference in anxiety values between the first and the second eyes (SMD: −0.40; 95% CI: −0.64, −0.16; *P*=0.001; Figure 5) with low heterogeneity (*I*^2^ = 6%). The anxiety value was lower in patients who underwent the second eye surgery. Analyses of the cooperation values of the patients graded by surgeons were also carried out and showed no difference between the first and second eyes (WMD: 0.35; 95% CI: −0.07, 0.76; *P*=0.10; Figure 6) with high heterogeneity (*I*^2^ = 91%).

### 3.4. Publication Bias

**Figure 7:**
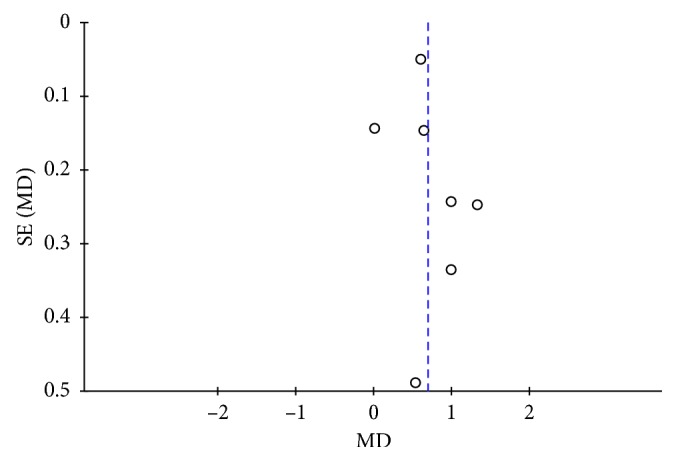
Funnel plot showing the publication bias of studies comparing pain scores shortly after the surgery.

Furthermore, an assessment of the publication bias was conducted for the outcome of pain score on the day of surgery under the local anesthesia and then presented as a funnel plot (Figure 7). No evidence of publication bias was observed. We also detected no publication bias (*P*=0.59) by the Egger test.

## 4. Discussion

Our meta-analysis showed that there was a significant difference in pain perception assessed shortly after phacoemulsification between the first and the second eye surgery, which meant that the patients felt more pain during the second eye surgery. The meta-analysis of the two studies confirmed that there was a substantial decline in anxiety values in the second eye surgery. This was likely to relate more with pain in the second eye, even if the results of three studies showed no variations in cooperation of the patients. What's more, on the first postoperative day, the meta-analysis of two studies showed that pain perception had no statistical difference between the first and second eyes.

Phacoemulsification is commonly accepted as one of the best surgeries for the treatment of cataract with the benefits of small incision, minimal induced astigmatism, as well as early visual rehabilitation [15]. The topical anesthesia is both a safe and an effective anesthesia methodology for clear corneal phacoemulsification cataract surgery with low and bearable pain [16]. However, there are certain patients who frequently encounter pain during phacoemulsification with topical anesthesia, while the pain perception does not necessarily have effects on the success of surgery [2]. A usual phenomenon proposes that the patients commonly complain of more pain or ocular discomfort when undergoing ophthalmic procedures in their second eye than in the first eye.

As reported by the first study conducted by Sharma et al., no substantial difference was observed between the first and the second eyes [11]. Subsequent to this, two studies by Hari-Kovacs et al. and Bardocci et al. also revealed similar results [5, 6]. On the other hand, recent studies have reported that the second eye experienced more pain than the first eye during phacoemulsification with local anesthesia [4, 12–14]. The probable reasons for these results could be anxiety before surgery, time of anesthetic drugs, and tolerance to anesthetic drugs, along with possible sympathetic irritation.

The result of present meta-analysis shows that patients have more pain in the second surgery, which is explained by the assumption that patients who have successfully undergone the first eye surgery are likely to have less anxiety and expect lower pain perception for the second eye surgery. However, few studies support the fact that less anxiety is likely to induce the analgesia rather than hyperalgesia [17]. As such, there is a need for us to perform further studies to examine the impact of anxiety on the intraoperative pain. Still, on the first postoperative day, there was no difference in pain perceptions between the first and the second eyes. This is likely to prove that immediate postoperative period is still before the peak amnesic effect of midazolam, in which patients are not capable of strictly comparing the pain values between surgeries, which is also followed by underestimating the pain scores of second surgery.

In the recent past, one trial on patients undergoing laser-assisted in situ keratomi (LASIK) revealed that they experience more pain during the second eye surgery [18]. Considering the same result as that of the phacoemulsification, we came up with speculations that whatever the eye surgery or anesthesia methodology used, there may have been some mechanisms under the phenomenon for the second eye to experience more pain during the surgery.

There were numbers of hypotheses considered to explain more pain in the second eye surgery. At first, the patients that had successfully undergone the first eye surgery were more probable to feel less anxiety while paying more attention to the pain perception during the second eye surgery [12, 14]. Secondly, the drug tolerance may occur due to the sedative and analgesic effect carried over from the first surgery in addition to the relatively long interval between the bilateral surgeries. Thirdly, the first eye surgery was regarded to give rise to sympathetic irritation making the contralateral eye prone to painful stimuli [12]. As proposed by one of the recent studies, a pain-related inflammatory chemokine, MCP-1, showed significant increase in aqueous humor in the second eye subsequent to the first eye cataract surgery, suggesting that there might have been a sympathetic ophthalmic type uveitis in the second eye prior to the first eye surgery to explain why the second eye surgery was more painful [19]. Preoperative administration of pranoprofen had been tested in reducing the pain during the second eye surgery. However, the specific mechanism is unclear [3]. All the hypotheses need to be confirmed by further investigation.

In consideration of the factors that had an association with pain during the surgery, Tan et al. showed that female patients tended to feel more pain during cataract surgery [20]. As well, Gombos et al. also reported that young patients as well as patients with higher cortisol and noradrenaline serum levels exhibited more sensitivity to pain during cataract surgery [21]. However, other studies reported no association between pain and age or gender [2, 22, 23]. Meanwhile, Rothschild et al. reported that surgery duration was sizably longer in the patients having high pain scores [24]. Patients experienced more pain in dominant-side cataract surgery than non-dominant-side cataract surgery under topical and intracameral anesthesia [25]. In recent studies, Yong et al. reported a relatively comprehensive study considering the factors associated with higher pain. These factors were regarded to include higher preoperative intraocular pressure, greater anterior chamber depth, and greater axial length [23]. Despite the fact that these factors were likely to be associated with experienced pain of patients during the surgery, whether they were the reasons for the second eye experiencing more pain is still necessitated further investigation.

In our meta-analysis, we encountered some limitations. Firstly, some studies did not use blind assessment in the methodology or bilateral eyes from an individual person were not available. Moreover, the interval time between two surgeries for some studies was not clearly stated. However, those problems did not strongly influence the overall effect of meta-analysis in pain because the results were quite consistent by sensitivity analysis of dropping the study with low quality. Secondly, the pain scores on the first postoperative day as well as the anxiety scores were only obtained from two studies that were likely to suggest the low reliability of the outcome. Therefore, further investigations are needed. Thirdly, the search strategy impacted on the number of included studies, since the search language was constrained only to English, possibly contributing to languages bias. However, we made use of specific analysis methodology to screen the studies and extract data with the consensus of discussion groups, in addition to assessing the maximum likely information in the studies. These approaches were effective in minimizing the likelihood of bias, thereby contributing to achieving reliable outcomes.

## 5. Conclusions

Summarily, our findings have clearly shown that patients perceived more pain in the second eye surgery than in the first, which is likely to be associated with various anxiety values between the first and second eye surgeries. Still, there is need for further studies to figure out the reasons for second eye surgery during phacoemulsification being more painful, so that we can take the preoperative administration to reduce the perceived pain during second eye cataract surgery.

## Figures and Tables

**Table 1:** Characteristics of included studies.

Author Year	Country	Number of eligible patients	Mean age (year)	Number of excluded patients	Consecutive recruitment	Blind method	Self-comparison	Interval of bilateral eye surgeries (week)	Time of assessing pain^*∗*^	Outcome of pain^*∗∗*^	Measure of pain	Local anesthesia	Local anesthesia drug	Additional anesthesia	Time of assessing anxiety^*∗*^	Outcome of anxiety^*∗∗*^	Measure of anxiety	Number of surgeons
Sharma et al. 2008 [11]	Australia	127	NA	2	Yes	NA	No	NA	1	No	VAS	Topical	1% amethocaine + 1% ropivacaine	—	1	No	VAS	1
Bardocci et al. 2011 [6]	Australia	73	75.2	8	Yes	NA	Yes	0.86–23.1 SD: 22	0	No	VAS	Topical	2% xylocaine	—	—	—	—	1
Ursea et al. 2011 [12]	USA	65	64	NA	Yes	Yes	Yes	1–32 SD: 6	0	Second	VAS	Topical	NA	—	0	First	APAIS/STAI	1
									1	No								
Hari-Kovacs et al. 2012 [5]	UK	187	76.5	13	Yes	NA	Yes	9.99–16.6	0	No	VAS	Topical	0.5% proximetacaine	Hydrodissection with lidocaine	—	—	—	1
Asian et al. 2013 [13]	Turkey	60	68.45/70.86	3	Yes	Yes	Yes	0–12	0	Second	VAS	Topical	0.5% proparacaine	Intracameral anesthesia with lidocaine	—	—	—	1
Jiang et al. 2015 [14]	China	159	67/69	8	NA	NA	No	NA	0	Second	VAS	Topical	2% lidocaine	—	0	No	VAS/STAI	1
Akkaya et al. 2016 [4]	Turkey	268	68.7	NA	Yes	Yes	Yes	0–12	0	Second	VAS	Sub-Tenon's	2% lidocaine + 0.75% bupivacaine	—	—	—	—	4
Zhang et al. 2018 [3]	China	38	66.6	NA	Yes	Yes	Yes	0–8	0	Second	VAS	Topical	NA	—	—	—	—	1

VAS: visual analogue scale; Amsterdam Preoperative Anxiety and Information Scale (APAIS); State-Trait Anxiety Scale (STAI); NA: not available. ^*∗*^Time of assessing pain and anxiety: 0 on the day of the surgery; 1 the day after the surgery. ^*∗∗*^Outcome of pain and anxiety: first: first eye is more painful (anxious); second: second eye is more painful (anxious); no: no difference.

**Table 2:** Score of quality of included studies.

	Representativeness of the cohort (consecutive recruit)	Selection of the second eye cohort^*∗*^	Ascertainment of exposure (anesthesia)	Demonstration of criteria for patients	Comparability^*∗∗*^	Assessment of outcome (blinding)	Same surgeon	Adequacy of follow-up of cohorts^*∗∗∗*^	Total
Sharma et al. [11]	1	0	1	1	0	0	1	1	5
Bardocci et al. [6]	1	1	1	1	2	0	1	0	7
Ursea et al. [12]	1	1	0	1	2	1	1	0	7
Hari-Kovacs et al. [5]	1	1	1	1	2	0	1	1	8
Asian et al. [13]	1	1	1	1	2	1	1	1	9
Jiang et al. [14]	0	0	1	1	1	0	1	1	5
Akkaya et al. [4]	1	1	1	1	2	1	0	0	7
Zhang et al. [3]	1	1	0	1	2	1	1	0	7

^*∗*^Demonstration of the interval of the first eye and second eye surgeries. ^*∗∗*^If the first eye and the second eye are self-comparison, score is 2. If patients' characteristics are the same between the first eye and the second eye but no self-comparison, score is 1. If the characteristics are not compared without self-comparison, score is 0. ^*∗∗∗*^Proportion of the excluded patients <10% and demonstration of excluded patients.

## Abbreviations

NOS: Newcastle-Ottawa Scale
ECCE: Extracapsular cataract extraction
WMD: Weighted mean difference
SMD: Standard mean difference
CI: Confidence interval
MD: Mean deviation
SD: Standard deviation
STAI: State-Trait Anxiety Inventory
APAIS: Amsterdam Preoperative Anxiety and Information Scale
VAS: Visual analogue scale
LASIK: Laser-assisted in situ keratomi
NA: Not available.
